# An innovation bootcamp model for developing youth-led HIV self-testing delivery strategies in Nigeria: post-designathon capacity building

**DOI:** 10.3389/fpubh.2024.1454304

**Published:** 2024-12-06

**Authors:** Ucheoma Nwaozuru, Chisom Obiezu-Umeh, Kadija M. Tahlil, Titilola Gbaja-Biamila, Rhonda BeLue, Ifeoma Idigbe, David Oladele, Donaldson Conserve, Collins Airhihenbuwa, Hong Xian, Adesola Z. Musa, Olufunto Olusanya, Temitope Ojo, Oliver Ezechi, Joseph D. Tucker, Juliet Iwelunmor

**Affiliations:** ^1^Department of Implementation Science, Wake Forest University School of Medicine, Winston-Salem, NC, United States; ^2^Department of Medical Social Sciences, Center for Dissemination and Implementation Science Feinberg School of Medicine, Northwestern University, Evanston, IL, United States; ^3^Department of Epidemiology, University of North Carolina at Chapel Hill, Chapel Hill, NC, United States; ^4^Nigerian Institute of Medical Research, Lagos, Nigeria; ^5^Division of Infectious Diseases, Washington University School of Medicine, St. Louis, MO, United States; ^6^Department of Public Health, The University of Texas at San Antonio, San Antonio, TX, United States; ^7^Milken Institute School of Public Health, George Washington University, Washington, DC, United States; ^8^School of Public Health, Georgia State University, Atlanta, GA, United States; ^9^College for Public Health and Social Justice, Saint Louis University, St. Louis, MO, United States; ^9^London School of Hygiene and Tropical Medicine, London, United Kingdom; ^10^Department of Medicine, University of North Carolina at Chapel Hill, Chapel Hill, NC, United States

**Keywords:** HIV self-testing, innovation bootcamp, participatory action research, young people, global health, Nigeria, co-creation, youth engagement

## Abstract

**Introduction:**

Many designathons, hackathons, and similar participatory events suffer from minimal training and support after the events. Responding to this need, we organized a health innovation bootcamp: an intensive, team-based apprenticeship training with research and entrepreneurial rigor among young people in Nigeria to develop HIV self-testing (HIVST) delivery strategies for Nigerian youth. The purpose of this paper was to describe an innovation bootcamp that aimed to develop HIVST delivery strategies for Nigerian youth.

**Methods:**

The four-week, in-person innovation bootcamp, informed by youth participatory action research and comprised a series of workshops, took place in Lagos, Nigeria. The goal was to build research and entrepreneurial capacities among young people to develop and implement HIVST strategies. A qualitative content analysis informed by an adapted World Health Organization’s HIVST delivery framework explored key elements of the proposed HIVST service delivery strategies developed at the bootcamp.

**Results:**

Twenty participants, aged 18–24 years, from five teams completed the innovation bootcamp. The five teams developed HIV service delivery strategies that included an element of repacking HIVST kits to make them more appealing to young people. Other strategies that emerged included leveraging community engagement platforms (e.g., vocational skills training and youth community events) to promote HIVST, and the use of reward-referral system to encourage HIVST uptake among young people. All strategies included ways to ensure privacy protection for recipients of the HIVST delivery package.

**Conclusion:**

This study demonstrated the feasibility and acceptability of the health innovation bootcamp model to create HIVST designed for and led by young people. This suggests a way to build capacity after participatory events to sustain youth-led research, which could have implications for post-designathon training.

## Introduction

Current HIV testing service delivery strategies in Nigeria often take top-down approaches, with a limited engagement of adolescents and young adults (AYA, 14–24 years), which often results in poor uptake of these interventions or programs (1). As a result, Nigeria lags in achieving the National and Global target of 95% HIV testing by 2030, especially among AYA (2, 3). HIV prevalence among young people in Nigeria (14–24 years) is estimated to be 3.5%, which is the highest in the West and Central African region (4). However, HIV testing remains low among this age group, with only an estimated 1 in 4 AYA ever testing for HIV (5). HIV self-testing (HIVST), a convenient and easy alternative to traditional facility-based HIV testing (6), may facilitate efforts to close the HIV testing gap among AYA. HIVST is a process whereby individuals collect their own oral fluid or blood specimen, perform a test, and interpret results at their own desired time or location (6, 7). Yet, expanding or scaling up HIVST among young people in resource-limited settings is poorly understood.

In recent years, there has been a growing interest in harnessing the capabilities of youth to generate novel solutions to public health problems that affect them (8, 9). This is because expert-driven approaches to solving public health challenges have been limited in addressing health challenges faced by young people (8). There is a push for intervention development and implementation processes to be appropriate, effective, and sustainable, and to reach the end-users, including youth (10). Youth participatory approaches offer potential solutions to these needs, as they are attractive vehicles to engage young people to spur innovations and address health issues that affect them. Youth participatory approaches have become an increasingly popular avenue to generate enthusiasm for innovation (8, 11, 12) and engage young people in solving pressing health problems that affect them (13, 14). One such participatory approach is an innovation bootcamp. This approach moves beyond tokenism and actively engages the end-user through capacity support and skills building.

This paper describes a four-week 4 Youth by Youth (4YBY) innovation bootcamp organized following the completion of a 72-hour designathon [more details about the designathon are provided here (15)] to expand HIVST among youth in Nigeria. While the 72-hour designathon provided a platform for early-stage ideation and development of the proposed solutions, the innovation bootcamp provided skills (including research and entrepreneurial training) necessary for the real-world implementation of HIVST delivery strategies among Nigerian youth. The innovation bootcamp brought together different communities of interest in a mutually supportive setting to accelerate proficiency for youth participants in research, including ethics training and building capacity in implementation delivery, storytelling, group learning, business model development, and field research. Our study aims to contribute to a stepwise approach to organizing innovation bootcamps among young people in resource-limited settings. By delineating the steps we took from idea generation to pilot fieldwork, we seek to provide valuable insights to researchers interested in using innovation bootcamps to expand health services designed for young people by young people.

## Methods

### Innovation bootcamp steps

The innovation bootcamp was part of the ITEST (Innovative Tools to Expand HIV Self-Testing) study, known as 4 Youth by Youth (4YBY). The goal of 4YBY is to increase the uptake of HIV testing and other preventative services among young people in Nigeria (16). The innovation bootcamp was held in Lagos, Nigeria, from May 6th to May 30th, 2019. The bootcamp was a four-week training program for young people to learn about research principles, entrepreneurship, and program management skills.

#### Step 1: identify and recruit teams

The top seven teams from the designathon, described here in more detail (15), were invited to register for the innovation bootcamp. Briefly, the 4YBY designathon was a 72-hour held between 29 and 31 March 2019, where 13 youth teams focused on designing solutions to promote uptake and awareness of HIVST among Nigerian youth. The deliverables from the designathon included a prototype of each teams’ HIVST kit service delivery and a presentation of their idea to a panel of judges. The invited teams who made it past the designathon, completed a pre-innovation bootcamp application form that included demographics (e.g., age, place of residence, the highest level of education, occupation) and project proposal (e.g., innovation name and components of HIVST delivery strategy). The teams ranged from two to five individuals from diverse educational and occupational backgrounds. One team facilitator was assigned to each team to mentor the teams and support them in achieving the boot camp deliverables as needed. The team facilitators were young people who had experience in HIV-related research and working with youth.

#### Step 2: organize pre-bootcamp activities

Preparation for the innovation bootcamp began 6 months before the event and was informed by a modified lean startup journey, which included (1) formation, (2) validation, and (3) growth (17, 18). Formation involved the teams further developing their innovation prototypes from the designathon. The validation phase involved the teams utilizing the “Build, Measure, Learn” feedback loop that took them through the journey of building a Minimum Viable Product (MVP) using ideas generated from the designathon. Lastly, growth involved working with the teams to identify ways to expand their ideas before the bootcamp. In addition, the research team and facilitators worked with the teams to identify the strengths, weaknesses, opportunities, and threats (SWOT analysis) of the teams’ proposed solutions from the designathon. Other pre-bootcamp activities included recruiting mentors for the instructional component of the bootcamp, recruiting judges for the project evaluations, and event planning logistics (e.g., acquisition of bootcamp venue, organizing transportation for participants, meals, and refreshments, scheduling field trips, and creating the schedule for the bootcamp). Mentors comprised representatives from public health research, communication, entrepreneurship, social innovation, design-thinking, youth engagement, social media management, product development, and marketing. The timing of the bootcamp was carefully selected to accommodate the participants’ schedules, given that most were university students. Food, transportation, and accommodation were provided for all bootcamp participants.

#### Step 3: accelerate bootcamp activities

The innovation bootcamp consisted of three main components: (1) instructional seminars and workshops, (2) field trips, and (3) iterative concept development supported by mentors and facilitators. The instructional seminars and workshops laid the foundation for participants to engage in research and turn their ideas from the designathon into HIVST service delivery strategies that could be implemented and tested for feasibility and preliminary efficacy. The concepts covered in the instructional seminars and workshops included creating strategic planning techniques, developing minimum viable products or services, startup development, implementation, planning budgets, and modeling costs, managing ethical issues around HIV and research, engaging with key community stakeholders, mastering storytelling techniques, and marketing strategies. Some of the workshops also included field trips.

The workshops were led by subject-matter experts who worked in Nigeria and had experience working with young people. The speakers included entrepreneurs, researchers, communication specialists, and community leaders. The schedule for the bootcamp is detailed in Table 1. At the completion of the innovation bootcamp, teams were expected to deliver three main items: (1) an innovation plan which describes the team’s approach and goals, (2) study protocol—a draft research protocol to be submitted to the Nigerian Institute of Medical Research to obtain ethical approval for the research component of their project, and (3) a pitch deck—slides to guide their 10-min presentations which consisted of information on team composition and strengths, key partners and community stakeholders, solution approach, cost model and pilot implementation budget, performance metrics, sustainability plans, and competitive advantage at the grand finale. Details of the three main deliverables are provided in Table 2.

**Table 1:** Innovation bootcamp program.

Topics	
Week 1	
Day 1	Introductions and recap of activities from the designathon
Day 2	Entrepreneurship and innovation mindset
Day 3	Research skills
Day 4	Community and stakeholder engagement
Day 5	Accounting, cost & logistics
Day 6	Site visits and fieldwork
Week 2	
Day 1	Questionnaire development and pilot-testing
Day 2	Data collection, analysis, & visualization
Day 3	Start-up development and storytelling
Day 4	Accounting, cost & logistics
Day 5	Site visits and fieldwork
Day 6	Pitch deck practice
Week 3	
	Off-site product development and meeting with team facilitators
Week 4	
Day 1	Offsite experience recap and presentations
Day 2	Offsite experience recap and presentations
Day 3	Pitch practice and feedback
Day 4	Innovation bootcamp finale

**Table 2:** Innovation bootcamp deliverables.

	Deliverables at the end of the four-week innovation bootcamp
1.	**Innovation Plan**
	*Develop a 4–6-page Innovation Plan that consists of the following contents:* About the team Short-term and long-term goals Significance Solution approach Key partners and community stakeholders Target audience and location Cost model Budget plan Tracking of performance and measuring of outcomes Project sustainability plan Competitive advantage Conclusion
2.	**10-min pitch deck**
	Develop a 10-min pitch deck. After this, jury members will take ten minutes to ask questions pertaining to the presentation. They will judge your project based on four criteria: feasibility of the innovation, user desirability, potential impact on young people in Nigeria, and teamwork.
3.	**Nigerian Institute of Medical Research Study Protocol**
	Using the Nigerian Institute of Medical Research (NIMR) Institutional Review Board (IRB) protocol template, teams will draft a study protocol which is a clear description of the study’s objectives, procedures, risks, benefits, recruitment, consent processes, and procedures to maintain confidentiality. NOTE: The study protocol will not affect the final scores. This is a preparatory and necessary process for the implementation phase.

Participants were given 3 weeks to complete their projects and have them ready for the final day pitch. The team were not required to have fully developed products or services for their HIVST service delivery strategies by the final day. Instead, the teams were required to accomplish the following: establish contacts with stakeholders who are integral to the pilot implementation of the HIVST service delivery strategies in their communities, complete an innovation plan and study protocol, and have a minimum viable product representing their HIVST service delivery innovation by the time of the presentation.

**Figure 1:**
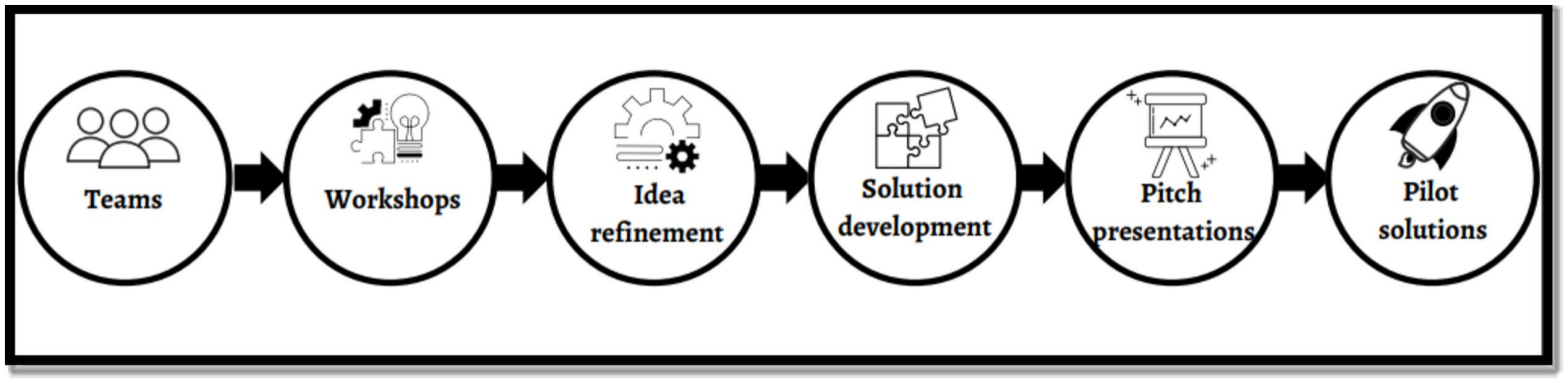
Innovation bootcamp flowchart.

Figure 1 shows the innovation bootcamp flowchart. This iterative process resulted in developing the final solutions presented to judges at the finale. The panel of judges comprised representatives from the Lagos State AIDS Control Agency, Google Nigeria, Nigerian Institute of Medical Research, United States Agency for International Development (USAID) Nigeria, and 4YBY youth ambassador. Following the finale, the next step was the pilot implementation of the solutions in the communities under the guidance of the research team.

#### Step 4: conduct bootcamp finale and final evaluation

The bootcamp finale was a day-long event where the teams pitched their proposals to a team of five judges and a broad audience (community stakeholders). The pitch involved a team presentation addressing the problem (e.g., low HIV testing uptake among Nigerian youth), the solution components of the HIVST service delivery strategy, team information and responsibilities, a plan for pilot implementation, and implementation outcome projections. The teams’ ideas and presentations were evaluated according to the four criteria, each accounting for 15 points of the total score: (1) Desirability: Does the innovation appeal to young people? Does it address the challenges (e.g., low-cost, accessible, confidential) that young people face when testing for HIV? (2) Impact: Will the innovation significantly support and champion young people to self-test for HIV? Is it possible to reach all young people in Nigeria when they are available? (3) Feasibility: Can the innovation be easy to implement? Are the resources available to execute the MVP? (4) Teamwork: How effective are the participants in working together as a team, sharing responsibilities, communicating with each other, and providing mutual support, with effective problem-solving and time management skills? The ranking of the teams was based on the average scores by the judges (60) and average innovation plan scores (40) scores from the research team. The final scores for each team can be found in Supplementary Appendix 1. Seed funding was provided to the teams toward the pilot of their proposed HIVST service delivery strategies: NGN 1,000,000 (≈2,850 USD) for first place, NGN 750,000 (≈2,140 USD) for second place, NGN 500,000 (≈1,420 USD) for third place, and NGN 250,000 (≈714 USD) for the fourth and fifth place positions. See Supplementary Appendix 2 and 3 on images from the innovation bootcamp workshop and innovation bootcamp finale pitch, respectively.

#### Step 5: post innovation bootcamp assessment and analysis

A post-innovation evaluation of the innovation bootcamp was conducted using a self-administered paper questionnaire comprised of open-ended questions to evaluate participants’ overall experience with the innovation bootcamp. The questions included were: “*What are your favorite part(s) of the innovation bootcamp?” “What are your least favorite part(s) of the innovation bootcamp?” and “What other topics do you think would be beneficial to include in the innovation bootcamp?”* Participants were recruited to complete the survey during the closing ceremony and while exiting the bootcamp venue the following day.

Prior to the end of the innovation bootcamp, two teams dropped out of the bootcamp due to non-compliance with contest rules and regulations. As a result, the bootcamp deliverables presented are based on the experience of the five teams that completed the bootcamp. The proposed HIVST service delivery strategies developed by bootcamp attendees were analyzed using qualitative content analysis based on an adapted World Health Organization’s (WHO) approach for the HIVST delivery framework (19). The framework uncovers: (a) “Who”—the agent who distributes the HIVST kits; (b) “What”—service and support tools are offered; (c) “Where”—where the HIVST kits are distributed; (d) “How”—how the HIVST kits are promoted; and (e) “Cost” of HIVST kit. Deliverables from the innovation bootcamp, such as the innovation plan and team pitch decks, were analyzed as additional data points. The open-ended questions from the survey are summarized as well (20).

### Ethical approval

Ethical approval for the ITEST/4YBY study was received from the Nigerian Institute of Medical Research Institutional Review Board, the Saint Louis University Institutional Review Board, and the University of North Carolina Institutional Review Board.

## Results

### Participant characteristics

The innovation bootcamp was completed by five teams with a total of 20 individuals. Two of the teams were from Lagos State, one from Enugu State, one from Oyo State, and one from Ondo State. The median team size was four participants (interquartile range: 3.5–4.5). Team 1 consisted of four males and one female, age range: 21–24 years; Team 2: two males and two females, age range: 19–24 years; Team 3: three males and one female, age range: 21–24 years; Team 4: two males and one female, age range: 18–21 years; and Team 5: three males and one female, age range: 19–23 years. Participants’ ages ranged from 18 to 24 years, with a median age of 22 years (interquartile range: 21–23.75). Fourteen participants (70%) were male, and 10 (50%) were from Lagos State. Eleven participants (55%) were college students, of which six were medical students, four were engineering majors, and one was an English major. Participants were from diverse professions: four health program coordinators, three National Youth Service Corps members (i.e., a mandatory service program for Nigerian college graduates), one computer programmer, and one graphic designer. The characteristics of the innovation bootcamp participants are presented in Table 3.

**Table 3:** Characteristics of participants at the innovation bootcamp, Nigeria 2019 (*N* = 20).

	*n* (%)
Age (years)	
18–21	9 (45%)
22–24	11 (55%)
Gender	
Female	6 (30%)
Male	14 (70%)
Profession	
University students	11 (55%)
Health program coordinators	4 (20%)
National youth service corps members	3 (15%)
Computer programmer	1 (5%)
Graphic designer	1 (5%)
Location (States)	
Lagos	10 (50%)
Ondo	4 (20%)
Oyo	3 (15%)
Enugu	3 (15%)

Data are number (*n*) and percent (%) of participants.

### Proposed HIVST service delivery strategies

Five viable HIVST service delivery strategies emerged at the end of the innovation bootcamp. Each proposed project centered around repackaging the HIVST using different potentially appealing packages to young people in various implementing communities. Table 4 presents the proposed HIVST service delivery strategies based on the WHO HIVST delivery framework (19).

**Table 4:** Service strategies for promoting (mobilization, testing, and linkage) HIV self-testing among young people aged 14–24 years in Nigeria.

Team Names	SMART	B-STAR	GENRA	BETERDOC	PHARMANAUT
WHO (distributes HIVST kits?)	The SMART ambassadors would generate community demand while distributing the HIVST kits.	The LUVBOX volunteers would generate community demands by targeting schools and artisan shops that hire young people.	The GENRA ambassadors would generate demands in the communities and university campuses.	The BETERDOC founders would generate demand in university residence halls.	The PHARMANAUT ambassadors would generate demand in their communities by targeting schools and community organizations.
WHAT (services and support tools are offered?)	Repackaging the HIVST kit to be more youth-friendly and rebranded the **“SMART Pack.”** The pack will include OraQuick HIVST kits, educational pamphlets, a referral coupon (for linkage to care and follow-up testing), and **SMART wristband** (to normalize HIV testing).	A self-care box called **“LUVBOX:”** repackaging the HIVST kits to include hygiene products (e.g., sanitary towels for females and beard care kit for males), sexual health products (including condoms), and USSD Pin code (for linkage to care and follow-up testing).	The HIVST kits will be readily accessible at pharmacies, retail shops, and social community events. The kits will also include **USSD code** for a step-by-step process on how to use the kit (which will be adapted in the local language “Igbo”) and the **on-call counselor** will be available for participants who test positive.	A BeterDoc pack will consist of an oral HIVST kit and an **access and referral pin code** that gives the customer access to BeterDoc website to **redeem exciting prizes** and opportunity to speak to a **trained counselor** (who can guide participants on how to conduct the test, understand the results, and link to appropriate youth-friendly services and care).	Repackaging the HIVST kit, to be called “IUNGO.” Iungo will include the HIVST kit, a **ticket to the IUNGO platform**, referral coupon (for linkage to care and follow-up testing), and educational pamphlets. The IUNGO platform aims to connect community members to **local skill acquisition programs** (e.g., basket weaving, tie-dye, etc.) based on the interest indicated by the participants.
WHERE (are HIVST kits distributed?)	Mobilization activities will be focused on young people between the ages of 14–24; the target population includes out-of-school youth and key populations.	Adolescents and young people aged 15–24 including students in the tertiary institution, in-school, and out-of-school youths	Young people aged 14–24 within university campuses and neighboring communities	Young people between the ages of 14–24 located within the university campus	Young people between the ages of 14–24 and focusing on youths in rural communities, tertiary institution students, and secondary school students
HOW (how are the HIVST kits promoted?)	**SMART Groove**: the SMART Pack will be promoted at social gatherings and outreach events. Participants who tested positive will be tracked and followed up through the **SMART Customer Relations System**, with the aid of the sales coupon referral cards issued during the SMART Grooves (the community social gatherings).	The LUVBOX will be promoted through short **films and storytelling on social media platforms** and in-person outreach events in schools and hard to reach communities.	Bili Vibes (meaning Bili Kam Bili which stands for Live and let Live in the Igbo language): will leverage the vibrant nature of the target population (young people) through a **novel social media app (including viral online challenges)** as well as fun **local community events** (e.g., soccer match and dancing competition)	BeterDoc will be marketed to students around the **university campus** through promotional flyers and booths.	The IUNGO platform will be promoted by **community campaigns**.
Cost (of the services provided)	Approximately 2.81 USD per pack	Approximately 1.39 USD per pack	Approximately 1.39 USD per pack	Approximately 2.81 USD per pack	Approximately 1.39 USD per pack

Statements in bold highlight the unique components for each model.

Team SMART aims to promote the distribution of HIVST kits through institutions, vocational centers, and social media platforms. The first-place proposal was by team “Smart.” Their approach involved using their “SMART Pack” to promote uptake of HIVST among young people at community centers through youth ambassadors called “SMART ambassadors.” The SMART pack is a rebranded and repackaged box for HIVST kits priced at 2.81 USD per pack. The pack will include OraQuick HIVST kits, HIV educational pamphlets, a referral coupon (e.g., for linkage to care and follow-up testing), and a SMART wristband (e.g., a plastic wristband to promote their SMART packs among young people). The target audience for the team were out-of-school youth and key populations.

The second-place proposal was by team “B-Star.” Their approach was to use the “LUVBOX” as a strategy to promote the uptake of HIVST testing among young people. The LUVBOX is a repackaged self-care box that includes OraQuick HIVST kits, hygiene products (e.g., sanitary towels for females and beard care kit for males), sexual health products (including condoms), and an unstructured supplementary service data (USSD) in code (e.g., for linkage to care and follow-up testing). The team proposed color-coded LUVBOXs, where male personal hygiene items will be included in the blue LUVBOX and female personal hygiene items in the pink LUVBOX. The repackaged LUVBOX was priced at 1.39 USD per pack. The target audience for the team was young people aged 15–24 years who were in school or out of school. The LUVBOX would be made available in supermarkets, online stores, mini-marts, pharmacies, neighborhood stores, and markets for easy accessibility in hard-to-reach areas. Team B-Star aimed to promote their HIVST service delivery strategy through short films and storytelling on social media platforms and in-person outreach events in schools and hard-to-reach communities.

The third-place proposal was from team “Genra.” Their approach involved using a program called “Bili Vibes,” a social media application that leverages community youth events, such as football matches, as a strategy to promote the uptake of HIVST among young people. The target audience for the team were young people aged 14–24 years within university campuses and neighboring communities. The HIVST kits would be available for interested individuals at community youth events, pharmacies, and retail shops. The HIVST kits were priced at 1.39 USD per pack. The kits would also include a USSD code for a step-by-step process on how to use the kit (e.g., which will be adapted in the local language “Igbo”), and an on-call counselor would be available for participants who test positive. Information about the HIVST kits would be included in the flyers promoting community center and school events.

The fourth-place proposal was from team “BeterDoc.” The “BeterDoc” strategy to promote the uptake of HIVST among young people involved using “BeterDoc Safety kits” that included an HIVST kit and leaflets that contained information on the location and phone numbers of the youth-friendly health centers in the community. The HIVST kits were priced at 2.81 USD per pack. The target audience for the team were young people aged 14–24 years within university campuses and neighboring communities. BeterDoc Safety kits would be promoted using flyers at community events and schools.

The fifth-place idea proposal was called “PharmaNaut.” Their approach was to use packaged HIVST kit, called “IUNGO BOX.” IUNGO BOX, priced at 1.39 USD per pack, would include the HIVST kit, a ticket to the IUNGO platform, referral coupon (e.g., for linkage to care and follow-up testing), and educational pamphlets. The IUNGO platform is an online marketplace with a list of local skill acquisition programs (e.g., basket weaving, tie-dye, etc.) for young people in the community. The target audience for the team were young people aged 14–24 years in schools (e.g., universities and high schools) and rural communities. PharmaNaut HIVST service delivery strategies would be promoted using flyers at community events and schools.

### Experience with the innovation bootcamp

Participants were asked to share their favorite aspects of the bootcamp. The fieldwork experience was noted as a favorite part of the innovation bootcamp for the majority of the participants. This may have been because participants had the opportunity to apply the knowledge, they were acquiring at the bootcamp in a real-world setting. Also, most of the participants stated that lessons on the purpose and use of MVPs and methods to set up milestones and measure progress were also relevant in building their entrepreneurial skills. Participants noted that the research plan development and communication training on storytelling for entrepreneurial venture promotion was also identified as the top favorite topics at the bootcamp. In addition, participants liked that the bootcamp provided a platform to connect with other young people passionate about a cause.

Some concerns were also raised about the bootcamp, such as time management and challenges with writing proper citations. The concept of time—the duration of sessions and time designated to work on their study—was mentioned by various participants. Some participants felt the classes were too long and indicated this was their least favorite part of the bootcamp. Other participants felt the structure of the bootcamp did not allow for sufficient time to work on their deliverables. In addition, some participants would have liked a class on making a reference list, perhaps during protocol development or writing their innovation (e.g., literature review). Some participants struggled with referencing their literature sources.

## Discussion

Our findings contribute to the current evidence on the relevance of active youth participation in closing the HIV testing gap among young people in resource-constrained settings like Nigeria. We extend the literature by using an innovation bootcamp model as a framework to provide experiential learning and active youth engagement in creating youth-led HIVST service delivery strategies for Nigerian youth. This approach emphasizes the value and feasibility of harnessing youth capabilities and creativity to create and implementation strategies. Such interventions are more likely to garner acceptance and be sustained as they reflect young people’s lived realities, needs, concerns, ideas, and solutions (21).

All five HIVST delivery strategies developed by youth participants prioritized serving both in-school and out-school Nigerian youth. This is particularly important with the increasing demand and uptake of HIVST among out-school youth that constitute underrepresented groups for HIV with complex sexual health needs (22, 23). It highlighted the teams’ awareness of the needs of the different groups of young people in Nigeria. This is in line with other studies that show that participatory design with the end-users ensures that outputs are culturally and logistically appropriate and feasible (24, 25). Another commonality in the HIVST service delivery strategies was focusing on repackaging the HIVST kits. This suggests that the appearance of HIVST kits is an important feature to consider increasing appeal among young people. The mode of repackaging varied across the teams. Some teams focused on exterior designs and some on the package content, including sexual health information pamphlets, personal hygiene materials, and referral coupons to youth-friendly health services for STI testing. The literature on health product packaging shows that the appearance of a health product impacts use, depending on if it elicits favorable or unfavorable traits (26). In this context, the teams sought to increase the discreteness and appeal of HIVST kits, which are important determinants of the uptake of sexual and reproductive health products among young people in SSA (27, 28). For instance, in a study among young women in Malawi, study participants suggested repackaging pre-exposure prophylaxis (PrEP) to look more youth-friendly and discrete to encourage uptake among sexually active young women (27). Discrete and appealing packaging of sexual health products could serve to mitigate concerns about stigmatization with the purchase of HIVST kits and encourage HIV testing (29).

In addition, the five proposed HIVST service delivery innovations included linkage to care component, which is often cited as a challenge with HIVST (30). Three teams proposed utilizing a referral coupon to youth-friendly health services, redeemable for entry into a raffle to win prizes. This is consistent with past research among young people documenting the benefits of incentives (e.g., monetary and nonmonetary) to encourage uptake of health services and behavior change (31–33). Although the effects of financial incentives often wane after it has been discontinued (34), it is beneficial to encourage the initial uptake and could be paired with other youth-friendly health promotion strategies. Two other teams proposed using USSD codes to encourage linkage to youth-friendly health services. USSD codes are widely used in SSA for mobile banking and to purchase mobile phone call time, data, and messages (35, 36). Therefore, we anticipate it would be an easy and familiar process among Nigerian youths (37).

Overall, the participants rated the innovation bootcamp highly, especially in the areas of research plan development, communication, and field trips to community stakeholders. In addition to enhancing their entrepreneurship and research skills, participants enjoyed the synergy and camaraderie with other young people that the innovation bootcamp provided. Despite the benefits of the innovation bootcamp in generating youth-led HIVST service delivery strategies, some limitations are worth mentioning. The innovation bootcamp training was limited to the top seven teams from the designathon previously organized by the research team. In addition, two teams dropped out before the end of the bootcamp We could not assess if the other teams that were not selected from the designathon would have successfully developed a viable HIVST service delivery strategy if exposed to the innovation bootcamp training.

Our findings have implications for research and practice. The innovation bootcamp approach goes beyond the traditional idea generation phase of other participatory approaches and focuses on developing and delivering intervention strategies. This model brings the best of both worlds by combining extensive entrepreneurship and research curricula typical of training courses with the fact-paced collaborative environments of designathons (38). This model can be a catalyst in developing robust and vibrant participatory interventions for public health solutions. From a research perspective, we exemplified how the innovation bootcamp model can help in providing a platform to engage young people to acquire skills to help develop and deliver health interventions. It provides a structured process to innovate in public health through active co-creation with community members by leveraging their knowledge. The HIVST service delivery strategies generated from the innovation bootcamp were being piloted to determine their feasibility and preliminary efficacy in promoting the uptake of HIVST among Nigerian youth. The findings from this study can also inform future interventions and policies that are more responsive to young people’s needs and build upon existing youth assets and capabilities, which are critical for community strengthening (39–41) and intervention sustainment.

## Conclusion

Our innovation bootcamp model was feasible and leveraged youth’s resourcefulness, capabilities, and resilience to generate youth-centered and youth-sensitive interventions to promote HIV testing. The innovation bootcamp led to the development of five youth-centered and participatory social innovations to promote the uptake of HIVST among young people in Nigeria. The emerging solutions from the bootcamp were piloted over 6 months, which examined the feasibility and efficacy of the solutions to promote uptake of HIVST and other sexually transmitted infection (STI) testing (42). The pilot study showed an increase in HIVST from 3 months compared with 6 months (20% vs. 90%; *p* < 0.001), as well as significant increases in testing for all four STIs: syphilis (5–48%), gonorrhea (5–43%), chlamydia (1–45%), and hepatitis B testing (14–55%) from baseline to the 6-month follow-up (42). The pilot study informed the development of an intervention bundle for further effectiveness trial in a randomized controlled trial. Overall, innovation bootcamp is a feasible approach to provide capacity support and research training for the implementation of participatory interventions among young people.

## Data Availability

The original contributions presented in the study are included in the article/Supplementary material, further inquiries can be directed to the corresponding author.
