# Supplementary material for: An innovation bootcamp model for developing youth-led HIV self-testing delivery strategies in Nigeria: post-designathon capacity building
Source: Front Public Health. 2024 Dec 6;12:1454304. doi: 10.3389/fpubh.2024.1454304 (PMC11659284; doi:10.3389/fpubh.2024.1454304)
Supplement: Supplementary file 1 [file Table_1.docx]

Appendix 1. Judges scores for the five teams at the innovation bootcamp Nigeria 2019

|  | **Judge #1** | **Judge #2** | **Judge #3** | **Judge #4** | **Judge #5** | **Total (100%)** |
| --- | --- | --- | --- | --- | --- | --- |
| **First Place Team** | | | | | | |
| Desirability (15) | 11 | 5 | 14 | 14 | 11 |  |
| Feasibility (15) | 10 | 6 | 13 | 11 | 12 |  |
| Impact (15) | 11 | 8 | 12 | 12 | 12 |  |
| Teamwork (15) | 12 | 10 | 14 | 13 | 13 |  |
| *Judges total* | 44 | 29 | 53 | 50 | 48 |  |
| *Average judges score (60%)* |  | | | | | 44.8% |
| Innovation plan (40%) | 32 | | | | | 32% |
| ***Grand Total*** |  | | | | | **76.8%** |
| **Second Place Team** | | | | | | |
| Desirability (15) | 12 | 13 | 11 | 11 | 12 |  |
| Feasibility (15) | 12 | 9 | 10 | 10 | 12 |  |
| Impact (15) | 12 | 11 | 9 | 10 | 12 |  |
| Teamwork (15) | 12 | 12 | 9 | 14 | 14 |  |
| *Judges total* | 48 | 45 | 39 | 45 | 50 |  |
| *Average judges score (60%)* |  | | | | | 45.4 |
| Innovation plan (40%) | 31 | | | | | 31% |
| ***Grand Total*** |  | | | | | **76.4%** |
| **Third Place Team** | | | | | | |
| Desirability (15) | 11 | 11 | 9 | 12 | 10 |  |
| Feasibility (15) | 12 | 9 | 8 | 6 | 12 |  |
| Impact (15) | 11 | 12 | 7 | 10 | 12 |  |
| Teamwork (15) | 12 | 14 | 10 | 12 | 13 |  |
| *Judges total* | 46 | 46 | 34 | 40 | 47 |  |
| *Average judges score (60%)* |  | | | | | 42.6% |
| Innovation plan (40%) | 32 | | | | | 32% |
| ***Grand Total*** |  | | | | | **74.6%** |
| **Fourth Place Team** | | | | | | |
| Desirability (15) | 11 | 8 | 11 | 13 | 11 |  |
| Feasibility (15) | 10 | 5 | 11 | 11 | 10 |  |
| Impact (15) | 10 | 10 | 11 | 12 | 13 |  |
| Teamwork (15) | 12 | 12 | 12 | 14 | 14 |  |
| *Judges total* | 43 | 35 | 45 | 50 | 48 |  |
| *Average judges score (60%)* |  | | | | | 44.2% |
| Innovation plan (40%) | 28 | | | | | 28% |
| ***Grand Total*** |  | | | | | **72.2%** |
| **Fifth Place Team** | | | | | | |
| Desirability (15) | 11 | 5 | 10 | 13 | 10 |  |
| Feasibility (15) | 10 | 8 | 9 | 11 | 13 |  |
| Impact (15) | 12 | 8 | 10 | 10 | 10 |  |
| Teamwork (15) | 12 | 8 | 9 | 14 | 12 |  |
| *Judges total* | 45 | 29 | 38 | 48 | 45 |  |
| *Average judges score (60%)* |  | | | | | 41% |
| Innovation plan (40%) | 22 | | | | | 22% |
| ***Grand Total*** |  | | | | | **63%** |

Note: The judges scored the teams' HIVST service delivery strategies based on the following domains: desirability, feasibility, impact, and teamwork. The maximum score that could be allotted for each domain was 15 points.


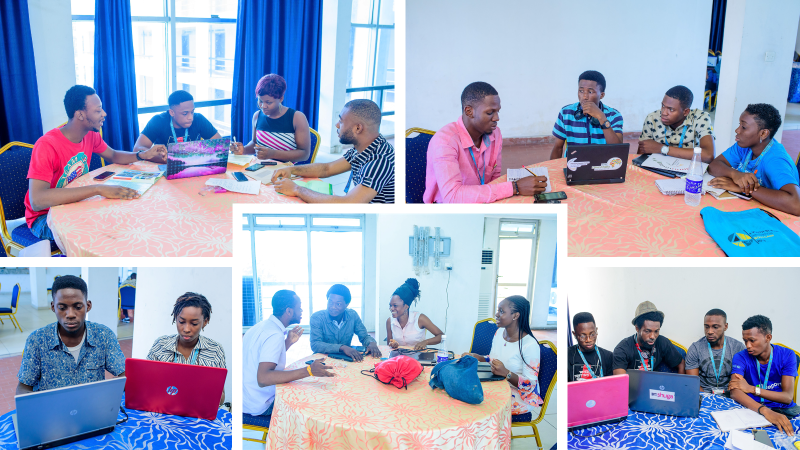
Appendix 2. Photographs from the innovation bootcamp Nigeria 2019 -The teams working together on their project


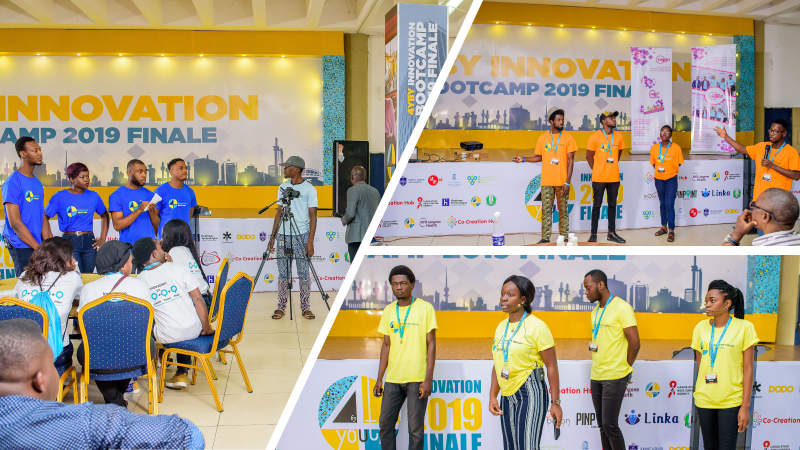
Appendix 3. Photographs from the innovation bootcamp Nigeria 2019 - Some teams presentation at the innovation bootcamp finale
